# Intense parenting: a qualitative study detailing the experiences of parenting children with complex care needs

**DOI:** 10.1186/s12887-015-0514-5

**Published:** 2015-11-26

**Authors:** Roberta L. Woodgate, Marie Edwards, Jacquie D. Ripat, Barbara Borton, Gina Rempel

**Affiliations:** College of Nursing, Faculty of Health Sciences, University of Manitoba, 89 Curry Place, Winnipeg, MB R3T 2N2 Canada; College of Rehabilitation Sciences, Faculty of Health Sciences, University of Manitoba, R106 - 771 McDermot Avenue, Winnipeg, MB R3E 0T6 Canada; Rehabilitation Centre for Children, 633 Wellington Crescent, Winnipeg, MB R3M 0A8 Canada; College of Medicine, Faculty of Health Sciences, University of Manitoba, 260 Brodie Centre - 727 McDermot Avenue, Winnipeg, MB R3E 3P5 Canada

**Keywords:** Parents, Children, Complex care needs, Qualitative research, Photovoice

## Abstract

**Background:**

Increased numbers of children with chronic illnesses and/or disabilities who have complex care needs are living at home. Along with the transfer of care to the home setting, parents assume the primary responsibility of their child’s complex care needs. Accordingly, it becomes even more important to understand the evolving roles and challenges faced by parents of children with complex care needs in order to better support them. The aim of this paper is to present research findings that add to our understanding of the roles parents assume in parenting their children with complex care needs.

**Methods:**

To arrive at a detailed and accurate understanding of families’ perspectives and experiences, the qualitative research design of ethnography was used. In total, 68 parents from 40 families were recruited. Data collection strategies included ethnographic methods of interviewing and photovoice. Several levels of analysis generated a sociocultural theme with subthemes representing how parents experienced raising children with complex care needs within the context of their life situations.

**Results:**

Intense parenting as the overarching theme refers to the extra efforts parents had to commit to in raising their children with complex care needs. Parenting was described as labour-intensive, requiring a readiness to provide care at any time. This left parents with minimal time for addressing any needs and tasks not associated with caring for their child. The main theme is supported by four sub-themes: 1) the good parent; 2) more than a nurse; 3) there’s just not enough; 4) it takes a toll on the health of parents.

**Conclusions:**

Overall, parents of children with complex care needs take on more roles as well as work more intensely at these roles than parents of healthy children. This, in turn, has led to the need for additional supports and resources for parents. However, to date, parents of children with complex care needs are still lacking adequate services and supports necessary to help them in their role of intense parenting. The findings sensitize professionals to the issues confronted by parents caring for children with complex care needs. Implications for further research and clinical practice are discussed.

## Background

Since the late 1980s, resilience and adaptation in the context of pediatric chronic illness and disability have become growing areas of focus [1]. Likewise, there has been a push towards family-centered care, which recognizes the important role of the family and parents in a child’s life [2, 3]. With these developments, there are increasing numbers of chronically and/or seriously ill children with complex care needs living and even dying at home [4–7]. Along with the transfer of care to the home setting, parents are required to assume the primary responsibility of their children’s complex care needs [8, 9].

With the added responsibilities and challenges, parents of children with complex care needs have reported role alterations. Most notable is the role of the nurse that requires parents to provide skilled nursing care in order to ensure their child’s well-being and survival [7, 10, 11].

Understandably, the increased caregiving demands experienced by parents can at times seem overwhelming for them even in the best of circumstances [12]. As parents take on complex care tasks, while balancing all other aspects of family life and work commitments, the complexity of the parental role and well-being of these caregivers is increasingly becoming a public health issue [3, 6, 8, 12, 13]. Increased caregiving demands are a serious risk for adverse psychosocial effects which calls for a better understanding of the consequences of caring for a child with complex care needs in today’s modern family [12].

The experience of caring for a child with complex care needs is seen as an evolving process, changing with the illness trajectory and as the child matures [9]. Parents have been shown to reflect changing roles and strategies of parenting [1, 10, 11, 14]. As more parents adapt their roles to care for greater numbers of children with complex care needs, it becomes even more important to understand their evolving roles and the challenges they face in order to better support them as they care for their children [7, 10]. The aim of this paper is to present research findings that further add to our understanding of the roles parents assume in parenting their children with complex care needs. The findings are derived from a longitudinal qualitative study that sought to extend our limited understanding of how the changing geographies of care influence the ways that Canadian families with children with complex care needs participate in everyday life [15]. The findings addressed in this paper specifically address the research question: “What is it like to raise a child with complex care needs?”.

## Methods

To arrive at a detailed and accurate understanding of families’ perspectives and experiences, the qualitative research design of ethnography was used. The inductive and evolving nature of ethnography afforded the opportunity for participants to make emic descriptions about what they think about and experience in raising a child with complex care needs in their own culture and social relations as parents [16–18].

### Setting and participants

This study took place in a major city in Canada. Families were recruited from a primary integrated health and social services community agency that provided services to children with complex care needs and disabilities that often required some form of technological support (e.g., mechanical ventilation, oxygen therapy). Ethical approval was obtained from the Education/Nursing Research Ethics Board at the University of Manitoba, Winnipeg, Canada. Prior to data collection, written informed consent was obtained from all parents. As well, on-going process consent was verbally obtained prior to each participant encounter considering this was a longitudinal study. We strived to ensure that ethical standards were maintained throughout the study, which included informing parents about confidentiality and the right to terminate their involvement in the study any time. All parents received a modest honorarium for their participation in the study.

To recruit participants a mix of purposive and snowball sampling techniques were used. We ended recruitment once redundancy was achieved at a final sample size of 68 parents (39 mothers and 29 fathers) from 40 families. The age range for parents was 22–56 years. Except for 13 parents, all were either married or in a relationship. Seventy-two percent of the study participants reported to be within the middle to higher income bracket. Thirty (75 %) of the families had at least one other child in addition to the child with the complex care needs. One family had two children with complex care needs for a total of 41 (12 females and 29 males) children with complex care needs. The children with complex care needs ranged in age between 6 months and 26 years with the mean age of 10 years at the time of the study. Cerebral palsy was the primary diagnosis for nine (22 %) of the children. Developmental disorders (e.g., global development delay), seizure disorders, terminal cancer, chronic lung disease, genetic disorders (e.g., Down syndrome), and congenital disorders were the primary diagnoses for the 32 remaining children. Table 1 provides a description of the technology used by the children.

**Table 1:** Technology used by children

	N
Types of Technology Used	
Feeding Tube	15
Mechanical Ventilation	6
Trachea	3
02 Therapy	6
Wheelchair	15
Suctioning	4
Saturation Monitor	4
Lifts	7
Walker/Stroller/Bike/Stander/Cart/Chair	22
Inhaler	4
Catheter	1
Special Bed	3
Braces	4
Speech Device	4
Hearing Device	4
Number of Technologies/Devices Used per child	
Zero	6
One	15
Two/three	9
Four/five	6
Six/seven	1
Eight/nine	4

### Data collection

Data collection was carried out by two research assistants. The research assistants were supervised and received extensive training in interviewing from the first author, who is an expert qualitative researcher. As the interviews were completed, the first author and research assistants reviewed the transcripts together, and feedback was provided as to what went well in the interview and any missed opportunities.

In order for us to arrive at a deeper understanding of the phenomenon under study and to better understand whether parents’ perspectives changed over time, parents were asked to take part in multiple in-depth, opened-ended interviews over the course of months [19]. We had planned to interview parents at three time points: at the start of their entry into the study, midway through, and prior to their exit from the study. However, not all parents were able to continue with either the second and/or third interview because of scheduling difficulties. In total, parents from 29 families took part in the second interview sessions and parents from 20 families participated in all three interview sessions. The open-ended questions and interview technique gave parents the opportunity to discuss what they considered important, to have greater control in the interview process, and to share information not anticipated by the researcher [19]. For the first interviews, the guide included questions about raising a child with complex care needs. For example, parents were asked to describe what a typical day was like for them, discuss how things were different since having to care for their child at home, and outline some of the challenges in raising a child with complex care needs. For the second and third interviews, additional questions based on the emerging data analysis were added to the interview guide.

In the first interview sessions parents were asked to draw an ecomap [20], starting with a circle that represented the parent and adding other circles as desired to represent people, activities, and places that are part of their lives. They were then instructed to draw lines between the circles to indicate the degree of bond between each person, activity, or place. During the second and third interviews, parents were asked to reflect on changes to their ecomaps. Rooted in family therapy and clinical family nursing practice with families, ecomaps are a graphic portrayal of social relationships and networks between individuals and spaces and surroundings [20]. Use of ecomaps provided additional information of the type of bonds and degree of attachments that parents experienced within their lives.

Complementing the second interview session was the participatory research method, photovoice, an innovative way for individuals to express their understanding and personal meanings of important issues [21, 22]. At the end of the first interview, the photovoice method was explained to each parent. Parents were given digital cameras and asked to take pictures of objects, people (if they obtained permission from them), places, or events that represented their everyday life, including the activities that they participated in. In the second interview, parents were asked to talk about their photographs by means of the SHOWeD method [22] which included asking them to describe what they felt was happening in each photograph and explain how it related to their lives. The meanings attached to the photographs provided further understanding of what is was like for the parents to raise a child with complex care needs.

The majority of the interviews were conducted in the families’ homes. Each interview lasted from 90–180 min. All interviews were digitally recorded and transcribed verbatim. Field notes were recorded to describe the interview context (e.g., parents’ non-verbal behaviours, communication processes).

### Data analysis

In keeping with the qualitative paradigm, data analysis occurred concurrently with data collection. Informing the data analysis process was all the data emerging from interviews, photographs, ecomaps, and field notes. Data analysis involved several iterative steps of analysis, congruent with ethnography: 1) isolating items or patterns referred to as cultural domains; 2) organizing domains by comparing, contrasting, and integrating items into higher-order patterns; 3) identifying attributes in each domain; and 4) discovering relationships among the domains [16–18]. The several levels of analysis generated a sociocultural main theme with sub-themes representing how parents experienced raising children with complex care needs within the context of their life situations. Throughout data analysis, attention was given to exploring similarities and differences between participants. Themes were refined after comparing data from the first set of interviews with the second and third set of interviews. During the second and third interviews preliminary interpretations were discussed with parents and this helped to uncover and lend support for the identified themes. Measures including prolonged engagement with participants and data, careful line-by-line analysis of the transcripts, and detailed memo writing were in place to enhance the methodological rigour of the study [23].

## Results

One overarching theme and four subthemes were identified that answered the research question. The overarching theme is intense parenting, with four sub-themes: 1) the good parent; 2) more than a nurse; 3) there’s just not enough; 4) it takes a toll on the health of parents. While there was variability with respect to each parent’s experience, the main theme and sub-themes emerged from the data of all parents in the study. There were no negative cases.

### Intense parenting

**Fig. 1:**
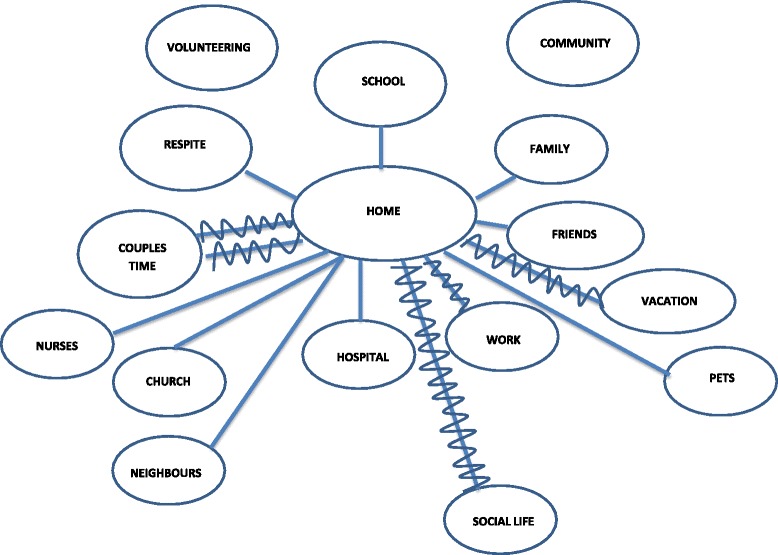
Ecomap-Father 7

This overarching theme refers to the extra efforts parents had to commit to in raising their children with complex care needs. In addition to the day-to-day challenges all parents experience in raising children, parents in our study also experienced challenges under the pressure of circumstances associated with the complex care needs of the child. Parenting was described as labour-intensive, requiring a readiness to provide care at any time. This left parents with minimal time for addressing any needs and activities (e.g., social life, vacation, and couple’s time) that are not associated with caring for their child as reinforced by this ecomap [see Fig. 1]. The parent that created this ecomap drew curvy lines for those relationships he defined as stressful and no lines in those situations where relationships were minimal or nonexistent.

A feature of intense parenting is that parents often faced the challenging effects of caring for children who remained fixed in a developmental stage or who struggled with moving on to the next developmental milestone. In short, the demands of parenting that coincide with each of the developmental stages were compounded for parents when children were unable to master one developmental stage and progress into the next. An example of this extended parenting occurs when the child may have the physical stature of an adolescent, while simultaneously requiring the physical care of an infant (dressing, feeding, and diaper changes). As one couple explained:

Mother: *She can’t walk, she can’t talk, she can’t eat, she has no fine motor skills, just sort of flailing around. Because she has CP she can’t articulate, and she is tube fed, in a wheelchair and diapered… Oh yeah. She can say mama… I’ve heard her say it. I know she can say it. She thinks this is very funny.* Father: *Yeah, ‘N’s’ eleven years old and I’m still waiting for dada or daddy.* Mother and Father 9.

There were also those cases where children made developmental gains but subsequently lost those gains, regressing to an earlier developmental stage and requiring parents to adapt to loss and change in function and ability. Parents at times felt disorganized and confused about the necessary steps that needed to be taken to address their child’s needs, specifically due to misplaced milestones and unique development requirements of their child.

Another characteristic of intense parenting involved parenting that was described as derailed. Parents shared examples of disordered and unsettled parenting experiences. One mother stated:

*No, it didn’t go smoothly because when we first got her out, it was nine months. She was back in the hospital within two days with a serious infection with (inaudible) and 24-hour feed, the equipment never worked, the pumps wouldn’t go. We were always at emergency at midnight. I was off. I never got any sleep.* Mother 9.

For most of the parents, intense parenting involved a *tag team*, or a partnership of intense parenting. Except for a few families where the mother assumed the primary caregiver role, the majority of parents in this study took turns and divided responsibilities and multiple tasks required for caring for their child with complex care needs. Working as a tag team facilitated their ability to cope with multiple physical, emotional, psychological, existential, and spiritual challenges that often arise from the time of the child’s birth. As one parent described it:

*Yeah, the best way is one of us stays here with ‘K’ and the other one takes ‘L’ out. Like last night I stayed here with ‘K’ and [my husband] took ‘L’ out to his grandmas to pick raspberries, and go to the park, and stuff. And sometimes I’ll just take ‘L’ and, and we’ll just go to the park and I’ll push him on the swing and stuff like that.* Mother 18.

For the majority of single parents, another family member (e.g., a grandparent) assumed the role of the other member of the tag team. Underlying the concept of tag team is the notion that the work or tasks that need to be done are so demanding that they require continuous effort, and there is little “down time.” While others sometimes assist parents (e.g., extended family, support services), the main source of energy for the family is time and the tag team. In many cases this results in intrusions with family engagements causing both physical and emotional strain for the parents.

The four sub-themes contributed to understanding the broad theme of intense parenting. Each sub-theme will be briefly explored.

### The good parent

Associated with intense parenting was the parents’ need to be seen as the “good parent.” For parents, being the “good parent” meant not only maintaining their child’s health and safety, but also ensuring that their child had a good life. It was reflected in parents constantly striving to meet their duties and obligations toward their child. In some instances, this involved going that extra mile and making sacrifices.

*I would never let him go without. And they do supply a lot of stuff to us and that, but it is just, you know, he has very sensitive skin, so I have to buy all [soap and lotions for sensitive skin]. I can’t just buy cheap stuff for him, it has to be the seven, eight dollars stuff. Um, I can’t just use any kind of soap or he’ll break out, so I can’t just buy cheap soap for him, like, you know…. At the beginning we’ve always had, like when, when he first was sick, I was working and that. And then, that’s when I had to stop because it was stressful for me to be at work when he was in the hospital and that.* Mother 11.

Parents in this study felt great pride in how they cared for their child and it was important that others were aware of the work involved in parenting a child with complex care needs. It was important that others perceived them to be “good parents,” as reinforced by the following:

*She has to be put out, so as parents, you don’t want her to go through that, because she’s gone through enough operations, so we make sure that we brush her teeth, and do a good job, and she’s eighteen years old. Um, you feel pretty good when you go to the dentist and the dentist says, “Yeah, her teeth look pretty good.” So as a parent, that looking after her teeth um, that’s good, but you accept that responsibility of, you know, ‘E’ (mother of child) wants to make sure that ‘K’ (child) looks presentable outside. I mean I think ‘E’ has seen a lot of people with disabilities that look unkempt. Their hair has not been brushed and that, so when we look after ‘K’ we want to make sure that she looks like she’s being looked after. Her hair is brushed. She’s wearing nice clothes um, you know, but that’s just uh the, the role that we’ve taken on.* Father 10.

However, parents did not always feel that others saw them as “good parents.” For example, parents of a teenage son with behavioural and emotional challenges expressed how they felt others judged them when out in public with their son.

Mother: *Well, like nothing’s really visible. It’s not physical….* Father: *They just think he’s a misbehaved kid, or they think you’re not very good parents.* Mother: *Well, I mean, there are a lot of times people look at you and think, “Like, why are you holding that kid’s hands for?” But it’s a comfort level for him.* Mother and Father 21.

While parents learned and adapted to address the complex care needs of their child, it was clear that society and the environment did not understand or always support parents who were striving to be the “good parent” to their child.

Father: *It is funny in terms of the, whether it is family or I assume, in a sense, whether it’s the system, that ‘N’ is not the only fourteen year old incontinent child.* Mother: *There’s no place to change her.* Father: *There’s no places to change her, men’s rooms are pretty awful type things, women’s rooms are better.* Mother and Father 9.

Parents in this study nonetheless rarely blamed others for not being able to support them in their “good parenting” role, but rather saw it as a lack of understanding on the part of others.

### More than a nurse

Intense parenting involved parents taking on a variety of roles to meet the needs of their children: health care provider, case manager, student, teacher, detective, guard, and advocate. All parents in this study took on these seven roles to varying degrees throughout their children’s lives, regardless of their child’s age or care needs. While parents in this study acknowledged that all parents of children assume some aspects of these roles, they nonetheless felt that parents of children with complex care needs did so with more intensity and did so throughout their child’s life.

### The health care provider

Parents provided many examples of assuming the role of a health professional to maintain their child’s health and well-being. This included assuming the role of the nurse, even when their child was in hospital, as reinforced by the following excerpt:

*He gets his meds at nine in the morning, nine at night, um and all his appointments we’ve been going to, and there’s the feeding tube…Before I had that electrical pump, we were doing it just through the nose, so then they gave me a stethoscope and all sorts of supplies and stuff and I would check for placement in his stomach. And like even at the hospital, too, I did his vitals and everything, like I did lots for him to help out the nurses, like when I was there. I just kind of watched and I just started doing it because they would say, “Okay I’ll be right back in five minutes.” Well then you’d see the nurse walking by, and you know they were busy.* Mother 8.

While the role of the nurse was common throughout the parents’ narratives, it was not the only health professional role that parents assumed, as illustrated in this father’s comments:

*Yeah, we’ve been trained on quite a bit of things through, you know, homecare, and through the medical system, and through the specialists that we deal with. So, physio, we’ve been trained in physiotherapy and occupational therapy, we’ve been trained in uh, in catheters and, and any piece of equipment she’s got. We’ve been trained, you know, for oximeters. And so if we go to the hospital we can basically, we bring our own equipment usually and we’re the ones, and we’ll put the mask on for her BiPAP (breathing apparatus), and we will monitor the probe, and then, and all those things.* Father 17.

**Fig. 2:**
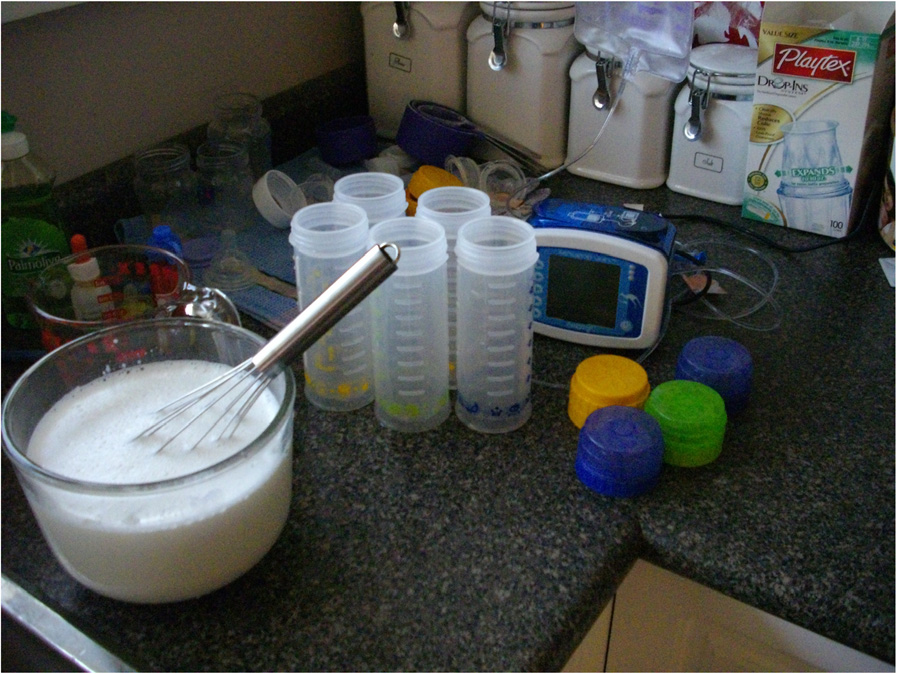
Health Supplies

The father in this excerpt commented on the knowledge he had gained about equipment used by his child. Equipment and/or supplies that are considered to be the norm of health care settings were also in many of the families’ homes as revealed by one of numerous photographs taken by parents [see Fig. 2].

Parents in this study discussed having to make informed decisions about medical treatment and care, weighing pros and cons of various courses of action, and considering potential consequences of decisions. In many cases making these decisions required acquiring considerable medical knowledge and training. As well, the training involved learning to adjust to the changing dynamics of the child’s condition. For example, a mother of an older adolescent explained that she and her husband were always tweaking her son’s medication because he is growing and changing. Although parents acknowledged the importance of assuming the multiple health professional roles, they voiced that acting as a health professional was challenging and “scary.”

### The case manager

In their role of case manager, parents became responsible for managing all aspects of their child’s daily life and care. On a daily basis parents described having to assess, prepare, implement, coordinate, monitor, and evaluate their child’s complex care routines and treatments. Most notably, the management of their child’s care involved extensive and advanced planning as reinforced by the following:

Mother: *I’m just saying that, that you can’t pre-plan enough here to realize what you’re going to do there, type thing, and it’s not that you can just suddenly, “Well, let’s take a quick tour over there ourselves just to see what it’s like,” and then bring ‘N’ (child). You can’t do that.* Father: *There are no proper facilities that you need in order to change ‘N’ and care for her* Mother and Father 9.

In an attempt to manage multiple challenging tasks many parents had established strict schedules and routines. There was so much planning and organizing involved in caring for a child with complex care needs that for some parents it was similar to running a business.

*I’m trying to, you know, um get better organized where because ‘M’ (son with complex care needs) is like, I mean he’s, he’s a child, but he’s like running a business in the sense that like there’s so much going on with him continuously. You know, you got surgeries and this and that, like there’s so much always that’s continuous that you need to really run him like a business in a sense. That’s because it’s continual for the rest of his life so. That’s how I look, like I don’t look at him as a business but -- do you know what I mean?… Everything’s scheduled for him, you know, to make sure that um, that he’s getting this and that… You have to go pick up his diapers, and make sure he has his medication, and make sure that he’s getting this many calories, and like it’s just, it’s continually every single day.* Mother 31.

Planning any event beyond what was scheduled in the child’s daily care plan involved considerable work on the parents’ part. At times, parents would forgo special outings or events, including holidays, because of the work involved in planning and undertaking such events.

### The student

In assuming the role of student, parents of children with complex care needs were engaged in a continuous learning process in order to educate themselves about their child and the child’s condition, care, and treatment options, as well as about the ways to cope with the daily requirements and challenges. From the moment parents were made aware of their child’s condition, they became students. As one father stated:

*He just looked at us one day and said, “I think she’s going to go home with you and you should start making plans.” You start learning. That was the first thing, learning about spina bifida.* Father 17.

Parental roles as students further intensified when they were faced with having to make life or death decisions. Staying informed was critical to making informed decisions related to their children.

Parents maintained their role of students as their children matured – they were always learning. As their children grew, there were many new aspects to learn about, including new treatments and/or services, changes experienced by their child, and navigating new systems (e.g., education system).

As students, parents gained knowledge and understanding through a variety of sources including their own trial and error experiences, the experiences of other parents of children with complex care needs, the internet, and professionals from the health, social service, and educational systems. Most importantly, parents gained understanding directly from their child with complex care needs. In sharing his experience of gaining knowledge and understanding, a father of an adolescent child noted:

*We have learned so much about ourselves. I would rather not have learned those lessons. I would far rather wish things had been otherwise, but ‘N’ is who he is. He is basically a happy child, we are very thankful for that. He has got his throwing up and everything under control. He does enjoy a lot of his aspects of his life, and it’s been educational to see that a person in his condition does have a life, he does, he really does. It’s not the life we would have wished for him, but it is the life he’s got, and it’s the life we’ve got, and we can live with that.* Father14.

### The teacher

Parents of children with complex care needs combined the role of being a student with that of a teacher. In their role of student they became knowledgeable about their child’s unique condition, symptoms, and treatment practices through extensive research as well as first-hand experience. As such, parents knew their child best and thus became a key source of information about their child. Parents were eager to provide guidance to respite workers caring for their child and share their knowledge with their children’s physicians and health care team.

*Especially for rare disorders, the doctors I think need to listen to the parents more,’cause the parents are doing all the research. ‘L’ (wife) went on and did so much research, talked to all the parents who’ve gone through things. At one time she recommended one of the new drugs to the doctors. The doctors never heard of it or had no idea about it, but she knew other parents who had success with [this drug]. She’s informing them.* Father 18.

Parents also noted that they helped educate their extended family, friends, and the wider community, about their child’s condition and needs. For example, a mother of a school-age child explained her role as an educator of her community in the following way:

*‘D’ (child) is our number one, that, nothing surpasses that. But then… the community is so important. Letting people know, like I’ll have people come up in the grocery store or wherever, and somebody will ask and we always try and answer questions about ‘D’. But the more knowledge people have uh, the better.* Mother 17.

Parents also found it was important to share their knowledge in parenting their children with complex care needs with other parents in similar circumstances as they felt it would help them deal with the challenges.

### The detective

Assuming the role of detective involved parents figuring out various aspects of their child and child’s care. For example, one mother of a child who was blind and hearing impaired described coming to understand that her child was blind:

*But he suddenly started to play and I just realized, I smiled right at this child and he doesn’t smile back unless I touch him or tickle him. But I can look right in his face and smile and I get no response. And he doesn’t reach out for any of his toys anymore, and uh, and so I, and so I sort of flicked my fingers in his face like this, and I got no response.* Mother 18.

While various tools and resources were available to help parents care for their children, the tools and resources were not always clear-cut or universal, so learning about them further required uncovering and investigative work on the part of the parents. A father of a school-age child explained:

*When people ask me now about, you know, what about raising children, I go, I don’t know how to raise a traditional child, because all I know is the one we had was very unique. But yeah, so that – it was, it was scary. We were given a lot of tools, but a lot of it we had to figure out ourselves.* Father 17.

Parents in this study shared examples of having to figure out how to make the technology and equipment that their child required work best for their child and their family. They explained that they would try various types of technology and treatments or medical procedures multiple times to find the ones that worked best for their child, and they were not always successful despite their efforts.

Parents sought to understand their children’s thoughts, feelings, and behaviours in order to be able to provide their children with the best care possible. In situations when parents were unable to figure out or understand their child, parents felt stressed and uncertain about their abilities to parent.

*You know he’s got a stomach ache, but what is it? Is it because he ate something wrong or, or he had the flu, or is it a pain, or is it, you know? And that’s the frustrating part in not being able to get that.* Father 24.

### The guard

To ensure their children’s safety and well-being, parents assumed the role of a guard which involved watching over and protecting their children. Part of being the guard involved parents monitoring their child’s health status as noted by the following:

*He was good all weekend and then Saturday he just started coughing all night…Yeah, I had to take him the hospital to be, just to be sure that it was nothing serious. They just said it was, you know, a little bug and that it would go away in a couple, a few days.* Father 4.

There was also the monitoring of procedures or treatments that children had to go through as this mother of a preschool child described:

*A lot of kids with neurological needs and special needs, you know, once they get really worked up it takes a long time to calm them, and yeah, I was pacing the hall. I could hear him screaming for like just about a half an hour. I could hear him screaming and just screaming…. They were just putting the probes on him and I finally came and banged on the door… and said, “Open it.” And I was just like, “Okay, this is way longer than a little while. He’s never going to calm him down, let me in there with my child.” I’m usually a fairly calm person, but I have to say that riled me up quite a bit, and uh, you know, I came in and then I calmed him* Mother 18.

Watching over their child’s psychosocial well-being was also important to parents and included monitoring how others responded to their child and protecting the child from inappropriate reactions or unwanted attention from others. Parents would try to avoid those situations that make their children feel uncomfortable. The role of the guard was hard to relinquish even for short periods and hence, resulted in parents staying close to their child within the home setting. Parents who were contemplating respite were reluctant to use it as they were uncertain if their child could be protected, resulting in parents missing out on social activities.

Similar to the other roles, while acting as a guard involved considerable work, it also helped to empower parents as reinforced by this husband as he spoke about his wife:

*She’s was very timid, and now she’s like a lioness guarding her cub. You don’t want to mess with her. And I guess you find, you find an inner strength that you never knew existed, and cause he’s (referring to their child with complex care needs), this is all we know. Like I, I don’t know how many times we’ve heard, I don’t know how you guys can do that, you know. People really respect us and admire us for what we do, and it is just all we know.* Father 7.

### The advocate

As advocates for their children with complex care needs, parents stood up for them and made sure that their children’s needs were met and their interests and self-worth respected. As one mother stated:

*‘C’ (child) has apraxia (short pause). This is ‘C’s’ road to walk and I can guide her along as best I can, but I can’t take it away. I have to make sure that she is everything that she can possibly be, but I have to protect her, and I have to advocate for her, and teach people how to treat her* Mother 29.

Parents’ role as an advocate was closely connected to their roles of students and educators. Gaining the necessary knowledge and developing the courage to stand up for the needs of their child were part of the same process for many of these parents. For example, a mother of a school-age child explained:

*You have to pull your boot straps up, and you’ve got to go online, and you’ve got to go to the library. You’ve got to do all this and then I had to learn to stand up for myself, which is huge. Not just myself, I had to learn to stand up for my kids. You have to become, um, what – their advocate. You have to learn, you know, that there’s nice ways to get things and to get what you want and then there’s a time when you have to stand up to a nurse and say, “Look, you have no right to treat me this way.”* Mother 25.

Many parents believed that without them advocating for their children, necessary resources and services would not be available for their child. For example, families shared experiences of having to fight to access certain equipment that, from their perspectives, helped meet their children’s needs and ensure their child’s safety. This included parents standing up against their child’s health care providers in situations when they did not agree. One mother of a school-age child, in sharing her experience of not agreeing with her child’s health care providers’ assessment of a situation, stated:

*And then we got into it with the hospital because we said, “Until you can tell me my kid’s safe, you’re not getting the machine (portable suctioning machine) back.” And they said, “Well there are other kids that need it.” I said, “Yeah, you tell me my kid’s safe, then you can have the machine back, but I’m not compromising my child.”* Mother 19.

The parents had to advocate for their children in a variety of settings including educational and health settings, but also in public and community spaces. Parents reinforced that if they did not advocate on a daily basis, nothing would get done in relation to their child.

Parents assumed the role of the advocate even when taking on such a role was not comfortable or was not in keeping with the way they usually acted.

*You know, like if you don’t try to go through the right channels or speak to the right people and, or the right resources, then, you know, then you can’t sit and complain or, you know, be frustrated… I’ve had to develop it (the role of the advocate) with ‘N’ (child with complex care needs). Um, like I have social anxiety, but with ‘N’ I try to push through and speak up.* Mother 7.

Then there were those parents who felt secure advocating in the public realm. Such parents were able to share their stories through various forms of the media (e.g., radio, television, magazines) and by contacting political representatives, like this father:

*Then we have the other things that exist in our society such as politics, and I think both of us have become more interested. We’ve actually even met with political figures and we’ve sent advocacy letters on things that relate to ‘D’ (child with complex care needs), but also I’ve become much more aware of the need to, to do these things. So, I’ve been sending letters to federal politicians about work-related things and other stuff.* Father 14.

Though all parents in this study took on these seven roles throughout their children’s lives, there were instances when certain roles were more prominent than others. For example, when their child experienced an illness exacerbation requiring hospitalization, the role of the guard became prominent with parents, especially watching over their child’s health status and care by health care providers. Another example involved key transitional periods such as their child’s transition from pediatric to adult care services or their child entering the school system. During transitional periods, parents needed to be educators and advocates in order to ensure those new to their child understood the child and his/her needs and that the necessary services were in place to meet the child’s needs. While fathers and mothers took part in all the roles, mothers more often than fathers assumed the health care provider and case manager roles.

### There’s just not enough

While parents acknowledged receiving a variety of services and supports, they nonetheless expressed that, for the most part, the services offered usually fell short of what was required to help them parent their child with complex care needs.

*Well, the support system just isn’t there for us, for one family to cope with a special needs kid by themselves, just a mom and a dad. And even if you’ve got other kids that aren’t special needs it’s, you know… They’re just, there’s just not enough. They’re like, you know, fine, come in and put a ramp system. That doesn’t help you at all when you, when you need to get out of the house for a couple hours or you’re going to burn it down, and believe me, there are days like that.* Father 6.

Parents expressed needing more of the services and supports currently offered to them and/or identified a number of services that they needed, but did not have access to. Table 2 describes the services and supports that parents most frequently identified that would help to ease the intense parenting.

**Table 2:** Identified services and supports

Services/Supports	Quotes
Financial Supports	*I know families they can’t make a financial go of it. We have most of our drugs covered and, and, you know the medical appointments are covered, but overall our expenses are so high and there’s no way of supplementing our income…. So they need to start looking at help for us.* Mother 5
° Examples: disability tax credit; tax free savings account for parents of children with complex care needs; drug benefit programs; funds for supplies (e.g., diapers, formula)	
Efficient Respite Services	*Evening, evening respite shifts. You know, even if it’s like once every two weeks just so that we have that night to, to go out and go catch a movie.* Mother 7
° Including: more respite hours; more flexibility with respite hours; access to respite when needed with no waiting period; qualified and consistent respite workers	
	Mother: *Respite. Of whatever community respite you can access.* Father: *You need to recharge your batteries and you’ll be better for yourself and for your child.* Mother and Father 34
Qualified Professionals and Support Workers who are: knowledgeable, caring, empathetic, respectful of parents’ knowledge, accessible, trustworthy, understanding of the child’s needs, good listeners	*What I would say is try to remember that you have no idea what someone else is going through, you have no idea, and you need to listen to parents. What I would say makes a difference between a great doctor and an okay doctor or nurse is just that ability to empathize professionally…* Mother 18
Family and Community Supports	*Before you might have to be the one who calls up and says, “Come over, I need you (friend) to come over. I want to talk to you. I need you”…. People might not, they might not be there for you unless you really just call them up and say, “I need to talk to you.”* Mother 18
° Examples: help from extended family and/or community members or organizations; understanding of the child and what it is like to be a parent of a child with complex care needs	
	*To find a good support system that you can fall back on and find out exactly what can help you out there.’Cause my wife has found so many different programs and organizations and stuff that can help out, and I think that’s the biggest thing.* Father 7
Integrated Knowledge Translation	*I would find someone who can give you all the information as to what’s available out there for your child but I, I still after five years have not found that person…. There’s no one, whether social work, or government level, or whatever, and it should be [someone] who can say to you “Okay, your child has lots of needs, let’s go through everything from physiotherapy to occupational therapy to whatever. Let’s look at all the resources that at some point you might like for your child, let’s say in the first five years, you know, music programs, swimming lessons, whatever.”* Mother and Father 3
° Examples: two-way information exchange between parents and those involved in the child’s care; keeping parents informed in a timely and ongoing manner; more information regarding navigating multiple systems	
	*Um, you know, it’s helpful to know what resources are available and I didn’t find that I had that information. I felt lost and like we were floundering for years. I just didn’t feel like we had those resources, didn’t know where to turn, you know*. Mother 27
Access to services/supports (including technology and/or equipment)	*Um, and Homecare made sure that we had all our equipment, Manitoba Home Nutrition made sure that we knew, you know, how to make formula and how to mix, you know, mix it properly, because we were making a big batch of it’cause that’s all he had. Um, I guess it was pretty smooth in that the services were trying to make sure we could take him home.* Mother 7
Improved Transportation	*…We had purchased a van and, but they’re not able to put in a lift and . . . to the van. What we have is just a portable, a portable ramp. Yeah, it’s very expensive to modify the van plus it’s the, the portable ramp, we were able to get a lighter one, but it’s not really very light, you know, so it’s, it’s for me, it’s harder . . . to be on my own to, it’s really hard to do it. And uh, a lift would have been much easier, you know, to take him.* Mother 16
° Examples: access to appropriate and affordable transportation; access to adequate parking spaces and loading zones	
	*They pay to have a wheelchair cab come and pick us up to take us to and from doctors’ appointments, which is really great, especially in the winter. Uh, especially when you’re dealing around the hospital’cause there’s never any parking, so it’s nice not to have to do the driving.* Mother 5
Accessible Spaces	[Looking at a picture they’ve taken] *This whole space is just barely enough to get her chair through. I have to come through here to get into the teen room, the teenage room. Parents put their bags; put their wheelchairs, everything blocking that.* Mother 5
° Examples: space that afford parents the ability to carry out their multiple parenting roles	
Promoting Self-Care in Parents	*Yeah. I’m going to be fifty. I don’t want to be dead at sixty or before sixty’cause stress kills, so I have to reduce that stress in my life. And so right now I’m dealing with people that are trying to help me recognize where the stresses are in my life and how are we going to reduce those, and I’m trying to get my wife on board, because um, I’m sure that she’s under the same kind of stresses. And so we have to decide whether we’re going to be on the same path or we’re going to have to decide to take separate paths,’cause I can’t, I can’t continue to do what I’m doing. That’s, that’s an obvious, so uh, we’re dealing with that at this time right now, between each other and so.* Father 10
° Examples: ensuring there is the time and opportunity for parents to take care of themselves (e.g., rest/relax, ‘me time,’ taking part in leisure activities, going for counselling, and so on).	

In some instances, parents reported relying on their own finances to get services and supports for their child (e.g., hiring a private speech therapist). Moreover, parents expressed that additional financial assistance would help them to meet the needs of their child. As well, parents expressed that services needed to be provided in a timelier manner. Parents described situations of waiting lengthy periods for a service and, in some instances the service was no longer needed when it was finally received. Parents reinforced the need for centralization or coordination of services offered to children with complex care needs. They expressed a need for more efficient systems that would better help parents meet the needs of their children. Quite often parents shared stories of a lack of coordination as this one father expressed:

*There is duplication between that agency and the school division. So there’s a social worker at the school and a social worker at that agency. There’s an occupational therapist at the school, an occupational therapist at the agency. And I’m like “Well, why does there have to be a duplication?” The problem is that there’s lots of room for conflicts to arise because of the fact that you have dual roles occurring.* Father 20.

### It takes a toll on the health of parents

Parents experienced physical and mental health difficulties as a result of intense parenting and the multiple roles that they had to assume in meeting their child’s needs. The sleepless nights and the overall lack of sleep that are often reported by parents of an infant were among the prevailing concerns and complaints for parents in this study, extending well past the infant stage for many families. Parents of adolescent-aged children discussed how they needed to stay alert to monitor the breathing of the child, change diapers throughout the night, or attend to night-time cries of the child, or how they stayed awake due to worry about the child. A mother of an older adolescent child explained:

*Well, it’s just he needs suction and then, you know, he wakes up and, or his diaper change…when you have a special needs son like that you can’t, you can’t really sleep, you know, unless there’s somebody there looking after him right. Because you don’t want to just sleep and then something might happen to him, you know.* Mother 16.

This lack of sleep over the years took an overall toll on the health of the parents. Parents also experienced chronic physical ailments as reinforced by the following:

*I have arthritis symptoms sometimes. I can’t open a jar from lifting her. And you put your back out and you’ve had your arms, you get physio and everything else, you get physical ailments. Like literally, I’m wearing out my joints.* Mother 9.

Regardless of the child’s complex care needs, all parents expressed experiencing high levels of stress at one time or another. One mother stated:

*Well it’s changed drastically. Um, uh you’re never prepared for that…. Um, it creates unbelievable amounts of stress, unbelievable amounts of anxiety. As a family it took a long time to get past that, “Hmm, am I going to wake up in the morning and she’s not going to be breathing anymore?”* Mother 3.

Parents were aware that the intense parenting would need to continue on for their child into adulthood and hence, feeling stressed and anxious about the child’s future was a consistent theme through many interviews. This included constant worry about their children’s future financial safety as they grew and transitioned to adult care.

The stress related to parenting a child with complex care needs was also compounded by others stressors experienced by parents, including other health issues. As one parent outlined:

*I think over the years it’s, it’s basically dragged me down where I, I’ve definitely dealt with physical problems… Just prior to [the holidays] I started to feel not well and then … I collapsed in the house, rushed, rushed me to the hospital. I went through the whole gamut of tests and that, go through my own doctor testing and everything, and physically everything’s okay. So, then you have to deal with the other aspect of it, the mental aspect of it… I have been diagnosed with anxiety, depression… You’re dealing with that and it’s not like just, it all of a sudden appeared, it’s just been gradually a progression where your body’s basically said, “Okay, we’ve had enough,” and it shuts down.* Father 10.

## Discussion

A dramatic shift in care responsibilities for families occurs with the corresponding increase in numbers of children with complex care needs living at home. Families do report positive impacts with this shift; indeed, parents in our study noted a number of rewards, which we will report on in a separate article. But it is also evident that the burden of care increases greatly as responsibilities extend beyond the usual care [9, 24–26]. Our study contributes to the existing research by further expanding on the work that is required of parents raising children with complex care needs from not only the perspectives of mothers, but also fathers. In contrast to Hays’ work on intense mothering that specifies intensive strategies of childrearing are the responsibility of mothers [27], our study revealed that participating fathers also shared the responsibilities. Mothers as well as fathers provided a picture of intense parenting that worked best within a tag team scenario in which parents take turns carrying out the necessary duties related to caring for their child with complex care needs [15]. Our finding could be explained by the fact that Hays did not focus on parents of children with complex care needs. Parents in our study discussed how intense parenting required roles that ranged from being a student to becoming an educator, and from acting as a guard to taking on roles of health care providers for their child. They explained that they often experienced derailed parenting involving misplaced milestones in their child’s development. Moreover, this study adds to our understanding of the impacts intense parenting has on parents, as well as discusses the resources that may support parents in their efforts to provide complex care for their children.

### More than a nurse

Parents in this study, as reported in previous research, assumed the role of the nurse [10, 11]. However, this was not the only role they assumed. Parents were expected to take on highly technical and/or specialized procedures normally expected of other health care professionals [28]. In order to ensure the health and well-being of their children, parents had to assume both the role of affectionate parent and professional caregiver. These roles may contradict each other due to the intrusive nature of the tasks that parents must perform [24, 29]. Finding a balance between the paradoxical roles can be a challenge in itself.

With the transfer of care to the home setting, parents of children with complex care needs have to take on a major role in the coordination and planning of their child’s care [28]. Research has revealed that families of children with complex care needs have been willing to take on the case management role, despite its added exhaustion, as it gives them a sense of control [14, 29]. Families have reported, however, that coordination and planning is sometimes extreme and incredibly burdensome, as was the case for the parents in this study [14, 29]. Parents in our study experienced the case manager role as intense, continuous, and energy consuming.

The high demands of caring for children with complex care needs resulted in parents becoming life-long learners. Parents in this study searched out information that would help them care for their children. Parents in other studies have also described themselves as “hungry for information” in a number of areas, including available services, financial counseling, and their child’s treatment [2, 14, 30]. The parents in this study engaged in the learning process that was at times stressful. In particular, they had to learn ways to maneuver numerous systems to ensure the individual needs and capabilities of their child were attended to. The learning process of parents was characterized by high emotional intensity due to the necessity to make informed decisions about their child’s health and well-being. Nonetheless, in addition to contributing to the parents’ ability to cope and manage their child’s care, research reinforces that acquiring information and new skills has been strongly linked with empowerment, as it increases parents’ confidence in providing necessary care [7, 29].

In addition to acquiring knowledge, parents had to ascertain what approaches would best meet the needs of their children. While they were given tools and resources, they still had to figure out ways to use them properly and effectively. The detective work became even more complex and difficult for parents who had trouble understanding their child’s behaviours.

Parents often know their child best because they are the primary caregivers and have an intimate relationship with them. Therefore, parents as teachers became a key source of information about their child. For the parents in this study, becoming a teacher to professionals, extended family, friends, the wider community, and health care professionals not only helped parents become a voice for their child, but also empowered them and other families in similar circumstances. Considering parents are the primary care providers and play key roles in the development of their children, it is important for professionals from the variety of systems to collaborate and share information with parents [3, 7, 30].

In the role of the guard, parents in this study discussed watching over their children to ensure their safety. While watching over their child and being present also helps create a trusting relationship between the parents and the child, it nonetheless involves parents missing out on relationships with family and friends. Other studies have shown that parents often temporarily restrict their social spheres and reject interactions with others during this time period of being there when nothing else can be done, in an attempt to further protect their child from physical danger, emotional stress, hostility, or invasive curiosity of onlookers [9, 31]. This occurred both in the hospital and home setting, and often persists until the family feels confident in managing the care treatment, and their child is outside a self-defined threshold of medical or emotional fragility. Even after parents become confident in the caregiving, however, parents in our study continued to assume the role of the guard.

Dealing with the diagnosis of illness or disability can be a struggle for parents. Parents must come to accept the realities of the changes that will occur. In doing so, many parents come to understand the importance of being an advocate for their child with complex care needs [31, 32], as was the case for parents in this study. In the process of advocating for their children, parents became assertive, working hard to get their children the services needed and making sure they are accepted in their society [31]. Parents shared that they had to develop courage and learn to stand up for their child in difficult situations. For example, parents discussed having to confront the staff at a child’s school or nurses at the hospital regarding treatment of their child that they considered unacceptable. As noted by Pohlman, advocating for their child with complex care needs can at times be difficult for parents as they may experience an imbalance of power between themselves and health care professionals [33].

## Challenges faced by parents

With the transition of complex care systems from government supported health institutions to the home setting, the roles of parents have significantly intensified for mothers and fathers alike [34]. While the ability for parents to take on a number of diverse roles is a sign of their personal growth [29], the experience of parenting a child with complex care needs was associated with a number of challenges. First, despite doing all that they could to protect and nourish their child’s health and well-being, often at the loss of other “normal” life events and activities, parents nonetheless felt at times judged by professionals and other individuals in their child’s life. As previously noted, parents of children with complex care needs can feel constrained by a society that does not value their efforts [35].

The intense parenting also resulted in health challenges for parents in this study, including parents experiencing physical problems and increased stress, anxiety, and depression. An especially troubling finding was that lack of sleep was among the most common health problem reported by all parents. Meltzer and Mindell note that caring for an individual with chronic illness often requires a significant amount of night care, resulting in significant sleep disruption and deprivation on top of the already physically draining tasks during the day [36]. In another study, parents reported experiencing poor health, headaches, and overall exhaustion related to their child’s sleep problems and to the necessity to attend to their needs at night [37]. Moreover, a study that focused on examining the impacts on parents of raising a child with perinatal stroke found mothers of children with moderate or severe conditions appeared to be at higher risk for psychological concerns and tended to have increased symptoms of depression and poorer health-related quality of life [38]. Past research has also shown that while the health and well-being of their child is the primary concern, parents of children with complex care needs have expressed that at times some of the care giving and treatment responsibilities they must provide are too much, leading to a need for assistance [39]. Despite wanting to give up, parents sacrifice their emotional and physical well-being for the sake of their children and often refuse to withdraw from their caring tasks [40]. Likewise, parents in our study were emotionally committed to their children and feared being separated from them while entrusting the care with others who they may view as lacking ability to provide the same level of care as they do.

Finally, consistent with previous research [7, 8], parents did not have enough services or supports. Nonetheless, parents in our study did whatever they could to ensure their child was well cared for and had a good life. When possible, parents relied on their own finances to pay for certain services and supports for their child. Previous research has also revealed that in order for their child to participate in the opportunities available in daily life, parents will use their own finances to make up for shortfalls related to technology, equipment, and resources [8]. Bourke et al. note that children with complex care needs necessitate direct costs on families that include extra medical attention, equipment, technology, devices, medications, and specialized therapy services, as well additional costs such as costs related to modifications of the family home [8]. Moreover, parents as revealed in our study assume tasks and responsibilities normally carried out by professionals who receive a salary for their work. Taken together, these findings suggest that additional financial assistance for parents of children with complex care needs is warranted. Professionals should also seek out parents’ perspectives of what it means to them to be a “good” parent. As Hinds point out, professionals need to ask parents how they can help them to fulfill their role of the “good” parent and what they need to meet their child’s needs [41]. Bearing in mind that parents’ and children’s needs change overtime, there should be regular assessments of needs to ensure families are receiving the appropriate services and supports [2].

### Strengths and limitations

While previous research emphasized the care assumed by mothers parenting a child with complex care needs [9, 28, 42], a major strength of our study was that fathers, in addition to mothers, contributed greatly to the study’s themes. Another strength of the study was the use of multiple qualitative data collection strategies, which provided parents a powerful way to communicate and share perspectives, and engage in critical thought and reflection on parenting a child with complex care needs. Nonetheless, there are limitations. First, while this study added to our understanding of the multiple roles assumed by parents of children with complex care needs, further work is needed that details how the roles vary based on the type of complex care needs. Warranted are studies that focus on parents of children with specific or similar complex care needs. While parents provided qualitative descriptors of the influence that intense parenting had on their health, prospective longitudinal research that incorporates both qualitative and quantitative measures would result in a more thorough understanding of the changing health status of parents of children with complex care needs. Research involving parents with differing ethnic backgrounds may result in additional perspectives about the roles that parents assume.

## Conclusions

With the transition of complex care systems from government-supported health institutions to homecare, the roles and needs of parents have significantly increased and intensified. Overall, parents of children with complex care needs take on more roles and work more intensely at these roles than parents of healthy children. This, in turn, has led to the need for additional supports and resources for parents. However, to date, parents of children with complex care needs are still lacking adequate services and supports necessary to help them in their role of intense parenting.
